# Transgenic Rice Expressing *Ictb* and *FBP/Sbpase* Derived from Cyanobacteria Exhibits Enhanced Photosynthesis and Mesophyll Conductance to CO_2_

**DOI:** 10.1371/journal.pone.0140928

**Published:** 2015-10-21

**Authors:** Han Yu Gong, Yang Li, Gen Fang, Dao Heng Hu, Wen Bin Jin, Zhao Hai Wang, Yang Sheng Li

**Affiliations:** 1 State Key Laboratory for Hybrid Rice, College of Life Sciences, Wuhan University, Wuhan, China; 2 Engineering Research Centre for the Protection and Utilization of Bioresource in Ethnic Area of Southern China, South-Central University for Nationalities, Wuhan, China; Institute of Genetics and Developmental Biology, Chinese Academy of Sciences, CHINA

## Abstract

To find a way to promote the rate of carbon flux and further improve the photosynthetic rate in rice, two CO_2_-transporting and fixing relevant genes, *Ictb* and *FBP/Sbpase*, which were derived from cyanobacteria with the 35SCaMV promotor in the respective constructs, were transformed into rice. Three homologous transgenic groups with *Ictb*, *FBP/Sbpase* and the two genes combined were constructed in parallel, and the functional effects of these two genes were investigated by physiological, biochemical and leaf anatomy analyses. The results indicated that the mesophyll conductance and net photosynthetic rate were higher at approximately 10.5–36.8% and 13.5–34.6%, respectively, in the three groups but without any changes in leaf anatomy structure compared with wild type. Other physiological and biochemical parameters increased with the same trend in the three groups, which showed that the effect of FBP/SBPase on improving photosynthetic capacity was better than that of ICTB and that there was an additive effect in ICTB+FBP/SBPase. ICTB localized in the cytoplasm, whereas FBP/SBPase was successfully transported to the chloroplast. The two genes might show a synergistic interaction to promote carbon flow and the assimilation rate as a whole. The multigene transformation engineering and its potential utility for improving the photosynthetic capacity and yield in rice were discussed.

## Introduction

Rice (*Oryza sativa* L.) is a staple food for more than half of the world’s population [1]. The world’s population is projected to grow from the present seven billion to an estimated ten billion people by 2050, with the growth concentrated in rice-consuming and rice-producing regions of Asia, Africa and the Americas [1,2]. Thus, each remaining hectare will have to feed at least 43 (presently 27) people in Asia due to population growth and increasing urbanization [3]. Concomitantly, more extreme weather, a scarcity of water and environmental pollution could also exert adverse effects on rice production [4,5]. Thus, the rice yield will have to increase by at least 50% over the next 40 years to prevent the mass malnutrition of the 700 million Asians that currently rely on rice for more than 60% of their daily caloric intake [3], and photosynthesis has been a major target for improving plant productivity via crop biotechnology in recent years [6].

Previous studies have shown that more than 90% of the crop biomass is derived from photosynthetic products, and the enhancement of photosynthesis at the level of per leaf area increases yields [7–9]. However, conventional breeding practices may be approaching a ceiling effect [10]. Recently, transgenic technology has been widely used to manipulate photosynthesis by overexpressing particular exogenous genes or introducing new enzymes or pathways that can positively influence photosynthesis [11].

Cyanobacteria have been regarded as ideal model systems for studying fundamental biochemical processes such as oxygenic photosynthesis and carbon and nitrogen assimilation, and it offers a rich source of genes for plant genetic engineering and the improvement of photosynthetic CO_2_ fixation [12,13]. The notable advantage of introducing cyanobacterial genes into plants was illustrated clearly by Zurbriggen et al. [14]. In addition, cyanobacteria have been present on earth for 3.5 billion years [15], during which time, they have endured a changing gaseous environment in which CO_2_ has declined and O_2_ increased. This environmental change has imposed evolutionary pressure on the cyanobacteria to evolve strategies for effective photosynthetic CO_2-_concentrating mechanisms (CCMs) to improve carboxylation via their relatively inefficient Rubisco enzyme [16,17]. The CCM involves five *C*_i_ uptake systems to effectively pump bicarbonate as the major carbon source and concentrate CO_2_ up to 1000-fold around the active site of Rubisco [18], however, the majority of the higher plants that belong to the C_3_ group, including most crop plants, do not possess this ability.

In C_3_ plants, due to the low kinetic affinity for CO_2_ and high resistance in the CO_2_ diffusion pathway (liquid phase in mesophyll cells), the partial pressure of CO_2_ at the catalytic site of Rubisco (*C*_c_, chloroplast CO_2_ concentration) is usually not saturated for carboxylation and is the ultimate limiting factor for photosynthesis [19,20]. Several genes have been identified in cyanobacteria that could promote the transport of CO_2_ and photosynthetic carbon assimilation. One gene is *Ictb* (inorganic carbon transporter B), which is involved in HCO_3_^-^ accumulation in *Arabidopsis* and tobacco. Its expression showed positive effects on photosynthesis [21–23], although the detailed mechanisms suggested by Price et al. were unclear [13]. The second gene is *FBP/Sbpase* (fructose-1,6-bisphosphatase or sedoheptulose-1,7-bisphosphatase), which is a dual functional enzyme in cyanobacteria that can hydrolyze both FBP (fructose-1,6-bisphosphate) and SBP (sedoheptulose-1,7-bisphosphate) with almost equal specific activities in addition to directly targeting Rubisco [24]. *FBP/Sbpase* plays an important role in regulating carbon flow and the regeneration phase of RuBP (ribulose-1,5-bisphosphate) and in catalyzing the first irreversible reaction in the conversion of triose phosphates to sucrose [25], as revealed by an increasing photosynthetic rate in tobacco [26–30].

Photosynthesis in plants has been considered for decades to be limited by only two factors: the velocity of the diffusion of CO_2_ through stomata, and the capacity of the photosynthetic machinery to convert light energy to biochemical energy and fix CO_2_ into sugars [31]. To improve mesophyll conductance to CO_2_ (*g*_m_) and further boost photosynthesis and yields, our basic aim was that rice should be reengineered at the biochemical level in the Calvin cycle to combine with inorganic carbon transport routes simultaneously and to achieve the high-efficiency operation of these two major processes in mesophyll cells. Although transgenic plants with cyanobacterial genes have been generated over the last two decades [12], most of them concentrated on single gene that acting on only one site in the biochemical or substance-transporting route. Based on the original work from the laboratories of Kaplan (*Ictb*) and Shigeoka (*FBP/Sbpase*) illustrated above, and considering the results of Feng et al. [32] from our laboratory, overexpression of the *SBPase* gene can enhance photosynthesis and growth under salt stress conditions as well as tolerance to CO_2_ assimilation during high temperature stress. Lieman-Hurwitz et al. [23] illustrated that the *Ictb* gene could improve photosynthetic rates compared to wild type under limiting but not under saturating CO_2_ concentrations. These results inferred that only one gene related to the Calvin cycle or carbon diffusion had not a significant effect on the enhancement of photosynthesis under normal natural conditions and had a much smaller effect on the yield. To overcome these shortcomings, we focused our analysis on the complete functions of *Ictb* and *FBP/Sbpase* compared with their respective functions derived from cyanobacteria following their introduction into rice. We anticipated that they would act on inorganic carbon transport and carboxylation in the Calvin cycle, respectively, further increasing the rate of CO_2_ transportation and the carboxylation efficiency under natural field conditions, potentially providing significant information for researching photosynthesis and rice production.

## Materials and Methods

### Construct generation

Full-length *Ictb* (*dc14*) and the optimised coding sequence of *FBP/Sbpase* (D49680) (S1 File) derived from *Synechococcus elongatus* PCC 7942 were amplified by PCR using primers *Ictb*-f (5' cggggtaccatgactgtctggcaaactctgac 3'), *Ictb*-r (5' gagtctagactacattttttcgtctgaatgct 3') and *FS*-f (5' cggggtaccatggctcaatccaccacttccgag 3'), *FS*-r (5' gagtctagatcagccaagcaggctgtcgacaaagt 3') respectively. The products were cloned into vector pUC18-35S-*rbcs-nos* skeleton (Wuhan biorun bio-tech. Co., Ltd), and the sequences were verified and found to be identical. The amplified 35S-*rbcs-Ictb-nos* and 35S-*rbcs-FBP/Sbpase-nos* skeletons were digested with BamHI, SalI and EcoRI, BamHI respectively, then ligated into pCAMBIA1301 to make pCAMBIA1301{35S:*rbcs*:*Ictb*:*nos*} and pCAMBIA1301{35S:*rbcs*:*FBP/Sbpase*:*nos*} respectively, and the sequences were verified by sequencing.

As for *Ictb+FBP/Sbpase* construct, the amplified 35S-*rbcs-FBP/Sbpase-nos* skeletons were cut and ligated into pCAMBIA1301{35S:*rbcs*:*Ictb*:*nos*} to produce pCAMBIA1301{35S:*rbcs*:*FBP/Sbpase*:*nos*}{35S:*rbcs*:*Ictb*:*nos*}. The amplified 35S-*rbcs-nos* sequences from pUC18-35S-*rbcs-nos* skeleton were digested with EcoRI and HindIII, and ligated into pCAMBIA1301 to generate the empty construct. All the sequences were verified by sequencing and found to be identical. The two genes were under control of the CaMV 35S promotor which directs constitutive high-level transcription of the transgenes, and guided by chloroplastic transit signal sequence *rbcs* (ribulose bisphosphate carboxylase small subunit) and followed by *nos* termination sequences to provide single *Ictb* and *FBP/Sbpase* and binary (*Ictb*-*FBP/Sbpase*) gene expression recombinant plasmids.

### Generation of transgenic plants

**Fig 1:**
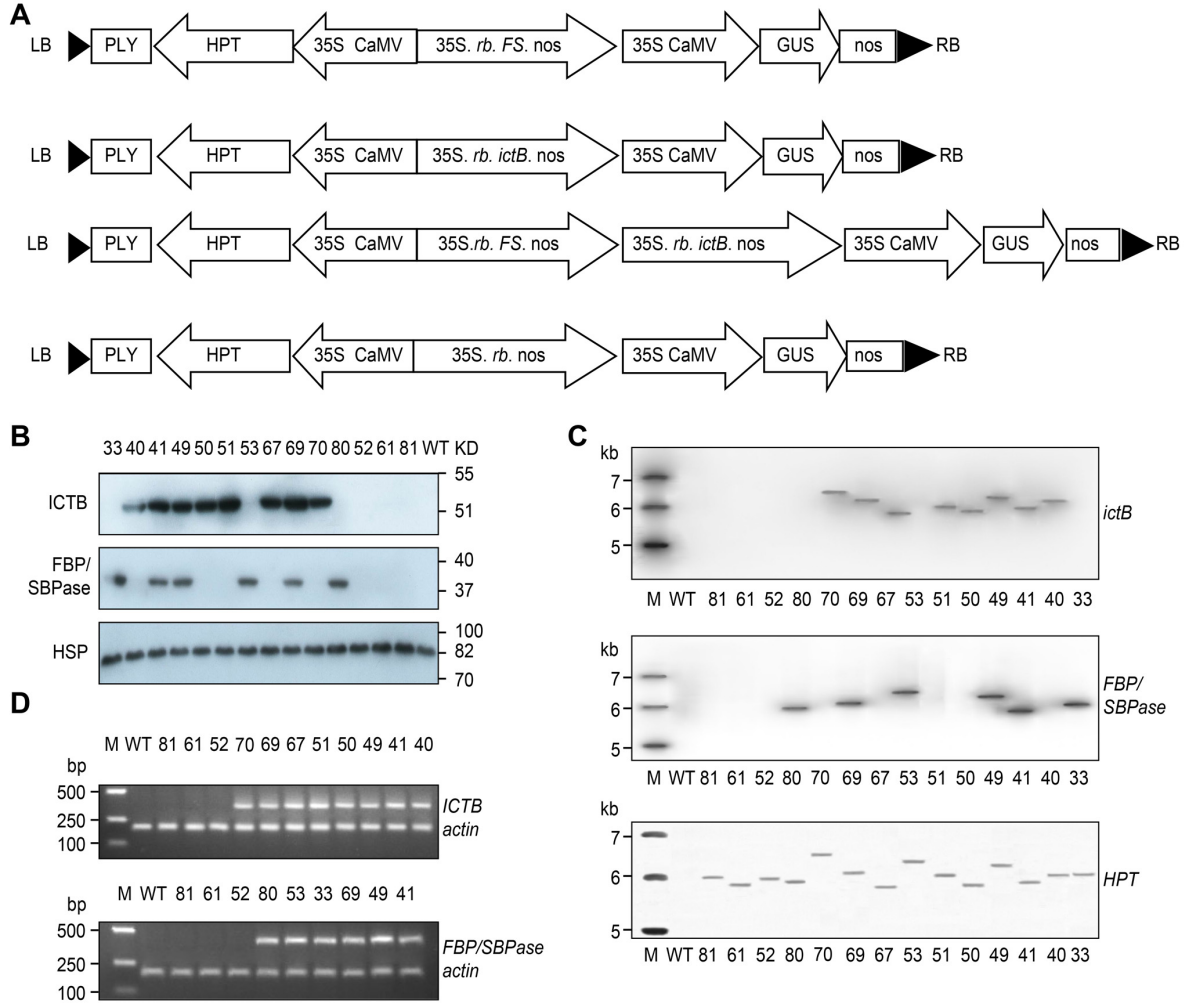
Production and selection of *Ictb* and *FBP/Sbpase* sense transgenic rice plants. **A.** Schematic representation of the T-DNA region of the pCAMBIA1301 binary vector. LB: left border, RB: right border. The sense construct contained full-length cyanobacterial *Ictb* and *FBP/Sbpase* cDNA driven by the CaMV 35S promoter, the nopaline synthase termination sequence and *rb*: chloroplastic transit signal sequence *rbcs* (ribulose bisphosphate carboxylase small subunit). **B**. Western blot analysis of transformants and WT rice. Ten micrograms of leaf protein samples from the newest fully mature leaves were separated by SDS-PAGE, and specific polyclonal antibodies were used to detect ICTB and FBP/SBPase proteins with the correct sizes. HSP (housekeeping protein) served as an internal control. WT: wild-type, *Oryza sativa* spp. *indica* vs. 9311. Marker: SM0671. **C.** Southern blot analysis showing the presence of the two target genes, *Ictb* and *FBP/Sbpase*, in the genomes of the transgenic lines. Genomic DNA was digested with BamH1 and hybridized with [α^32^P] dCTP-labeled specific probe. M: Marker, 1 Kb ladder. **D.** RT-PCR analysis of transgenic lines and wild-type based on the results of the western blot and southern blot analyses. Each lane represents a sample obtained from one individual line, and the 400-bp and 350-bp-long *Ictb* and *FBP/Sbpase* cDNA fragments, respectively, were obtained. *Actin* served as a loading control. M: Marker DS2000.

The single (*Ictb* and *FBP/Sbpase*, respectively) and binary (*Ictb*-*FBP/Sbpase*) gene expression recombinant plasmids, and empty construct plasmid were transformed into *Agrobacterium tumefaciens* EHA105 (Fig 1A). The *Oryza sativa* spp. *indica* vs. 9311 (wild-type) was transformed with these resultant plasmids using the standard *Agrobacterium*-mediated method as described previously [22,33]. Shoots were regenerated on selective medium containing hygromycin (50 mg L^–1^), and T_1_ plants were obtained by self-pollination of primary transformants (T_0_). T_6_ plants from 2013 were used in photosynthetic, physiological and biochemical analysis for three experimental replicates in this study.

### Plant growth conditions

The seeds of these transgenic plants were allowed to germinate on agar in the presence of 50 mgL^-1^ hygromycin, and the seeds of the wild type plants were allowed to germinate on agar in the absence of hygromycin. After growth for four weeks, 300 plants of each independent line in every group as an experimental unit was transferred to the same experimental field paddy and planted in sets of three. All of the experimental units were distributed randomly and grown under the same environmental conditions in Huashan country (exclusive transgenic rice experimental field of Wuhan university, Wuhan city, E114°31′, N30°32′). All of the measurements of physiological and biochemical parameters were conducted on flag leaves in the flowering stage after sowing for 97 days. The samples were selected randomly in the center of each line to obtain three experimental repeats for every group to avoid the edge effect of the rice population in the paddy. Data were derived from three independent replicates of each line in five groups.

### Protein Extraction and Western Blot Analysis

Approximately 0.1 g leaf tissue was harvested from the youngest fully expanded leaves and used for protein estimation and gel blot analysis. Protein estimation was determined according to the Bradford method using BSA as a standard. Equal amounts of proteins were separated by 12% SDS-PAGE and electroblotted onto nitrocellulose membranes and then probed using the specific polyclonal antibodies Anti-OsICTB and Anti-OsFBP/SBPase in 15 lines. The polyclonal antibodies were generated by immunizing healthy rabbits using the synthesized peptides of full-length ICTB (AAB08477) and FBP/SBPase (BAA08536) as antigens. The protein conjugations, immunizations, and antiserum purifications were performed by BPI (Beijing Protein Innovation Co., Ltd, Beijing, China), with an initial purity ≥ 90%. The entire generation process of antibody and the application of western blot detection using rice tissues were validated by the BPI company following the instructions of Li et al. [34]. Proteins were detected using horseradish peroxidase conjugated to the secondary antibody and Super-ECL® HRP chemiluminescence detection reagent (SuperSignal^®^ west Pico Chemiluminescent Substrate, Thermo Scientific, USA).

### Southern blot hybridization

Southern blot analysis was performed to verify the integration and copy number of the two targeted genes. The genomic DNA (30 μg) from WT and 14 transgenic lines was digested overnight with BamHI, separated in a 1% agarose gel and blotted onto a nylon transfer membrane (Nytran^®^ SPC, GE Healthcare, Life Sciences, Whatman^TM^, Amersham, UK). Probes with a size of 400 bp and 350 bp for the *Ictb* and *FBP/Sbpase* genes were prepared from PCR-amplified fragments using specific primers, respectively, and labeled with [ɑ^32^P] dCTP using the Random Primer DNA Labeling Kit Ver.2.0 (TaKaRa Biotechnology Co., Ltd, Dalian, China) following the manufacturer’s instructions. The filter was hybridized to the labeled probe at 65°C overnight, washed with 2×SSC, 0.1% SDS once at room temperature and twice at 65°C for 15 min each and finally washed twice with 0.1×SSC, 0.1% SDS at 65°C for 15 min each. The membrane was processed for autoradiography. All of the procedures for the hybridization were performed as described previously [35].

### RT-PCR analysis

Total RNA was extracted from fully expanded flag leaves of all of the lines using TRIzol (Invitrogen) and treated with DNase I following the manufacturer’s instructions. RT-PCR was performed to amplify the *Ictb* and *FBP/Sbpase* transcripts with specific primers: *Ictb-*1: 5′-cggttgccgacttcacctcacgg-3′, *Ictb*-2: 5′-ttgctgctgtcttcacgcccca-3′ and *FS-*1: 5′-ccgatcggtcgctatacgctgct-3′, *FS*-2: 5′-ggcgataccttcgggccactgca-3′ respectively. *Actin* transcript was also amplified as a control using the specific primers *actin-1*: 5′-gccttggcaatccacatc-3′ and *actin-2*: 5′-agcatgaagatcaaggtggtc-3′. The RT-PCR analysis was repeated three times in each line with similar results.

### Subcellular localization

The subcellular localization of the two genes was investigated by introducing a binary vector containing CaMV 35S:: *Ictb*-*GFP*, CaMV 35S:: *rbcs*-*Ictb*-*GFP* and CaMV 35S:: *rbcs*-*FBP/Sbpase*-*GFP*. The latter two constructs were almost identical to the initial transgenic binary vector with the only difference being the addition of *GFP* as an indicator in the rice mesophyll protoplasts. The protoplasts were prepared from 2-week-old etiolated rice seedlings that were grown hydroponically and used for transformation by the polyethylene glycol method as described by Cinelli et al. [36]. The GFP signal was observed using a FluoView FV1000 Confocal Laser Scanning Microscope (Olympus).

### Gas exchange, fluorescence measurements and the determination of mesophyll conductance

Leaf gas exchange and chlorophyll fluorescence were measured simultaneously using a Li-Cor 6400 portable photosynthesis open system (Li-Cor Inc., Lincoln, NE, USA). The measurements were performed using expanded flag leaves from 9:00 to 11:00 during the flowering stage under the conditions of a photosynthetic photon flux density (PPFD) of 1200 μmol m^-2^s^-1^ and *C*_a-c_ (ambient CO_2_ concentration in the cuvette) of 380 μmol mol^-1^ in the leaf chamber. The natural air temperature in the paddy field was 33–35°C, and the constant relative humidity (RH) was maintained at 80±5%. Photosynthetic parameters such as gas-exchange measurements, *F*_s_ (steady-state fluorescence), and F_m_^′^ (maximum fluorescence) were recorded after the measurement system remained stable. All of the experimental procedures illustrated above were performed according to Li et al. [37].

Measurements of *P*_n_/*C*_i_ (*P*_n_: net photosynthetic rate; *C*_i_: intercellular CO_2_ concentration) and light response (*P*_n_/PPFD) curves were conducted on the same leaves under the conditions described above, and the procedure was conducted according to Li et al. [37]. *C*_a-c_ was set as a series of 0, 50, 100, 200, 400, 600, 800 and 1000 μmol CO_2_ mol^-1^, while PPFD was maintained as 1200 μmol m^-2^s^-1^. The initial slope of the *P*_n_/*C*_c_ curves was calculated as the carboxylation efficiency (CE) when *C*_c_ was <200 μmol CO_2_ mol^-1^. Regarding the measurements of the light response curves, *C*_a-c_ was maintained at 380 μmol mol^-1^ in the leaf chamber, while PPFD was adjusted following a series of 0, 50, 100, 200, 400, 600, 800, 1000, 1200, 1400, 1600, 1800 and 2000 μmol photons m^-2^s^-1^ based on the methods of Li et al. [37].

The rate of mitochondrial respiration in the light (*R*_d_) and the CO_2_ compensation point related to *C*_i_ (Γ*) were measured according to Li et al. [37]. The intersection point of the *P*_n_-*C*_i_ curves was assessed at three different light intensities (150, 300 and 600 μmol m^-2^s^-1^), where *P*_n_ represented -*R*_d_, and *C*_i_ indicated Γ*. The measurements were conducted using the same flag leaves in the flowering stage from 0: 00 h to 4: 00 h in paddy fields [38–40]. At each PPFD detailed above, *C*_a-c_ was set as the series of 0, 25, 50, 80, and 100 μmol CO_2_ mol^-1^. To promote stomatal opening, the leaves were placed in the cuvette with a PPFD of 600 μmol photons m^-2^s^-1^ and a *C*_a-c_ of 100 μmol CO_2_ mol^-1^ for thirty minutes. The determination of Γ* and *R*_d_ was repeated three times and resulted in values that did not differ (P <0.05) according to Warren [41]. Γ* was corrected for the effects of temperature based on the temperature response equations reported by Bernacchi et al. [42].

Based on the gas exchange and chlorophyll fluorescence measurements, estimations of the total electron transport rate (*J*_T_) were calculated based on the procedures of Genty et al. [43] and Valentini et al. [44], and *g*_m_ and *C*_c_ were conducted using the method described by Harley et al. [45], as follows:

$$J_{\text{T}}=({F_{\text{m}}}^{'}-F_{\text{s}})/{F_{\text{m}}}^{'}\times \text{PPFD}\times \alpha_{leaf}\times \beta$$

$$g_{\text{m}}=\frac{P_{\text{n}}}{C_{\text{i}}-\frac{\Gamma^{*}\left[J_{\text{T}}+8\left(P_{\text{n}}+R_{\text{d}}\right)\right]}{J_{\text{T}}-4\left(P_{\text{n}}+R_{\text{d}}\right)}}$$

$$C_{\text{c}}=C_{\text{i}}-\frac{P_{\text{n}}}{g_{\text{m}}}$$

where α_leaf_ is the total leaf absorbance and is assumed to be 0.85 [37,46,47], and β represents the partitioning of the absorbed quanta between the two photosystems and is assumed to be 0.5 for C_3_ plants [37,48,49]. These equations have been commonly used to calculate the above values by Flexas et al. [19], Hassiotou et al. [49], and Vrábl et al. [50], among others.

### Determination of LMAs (leaf dry mass per area) and yield traits

LMA was measured using the same leaves that were used for the photosynthetic measurements. The leaf area was measured by a digital analysis of the images using ImageJ software (National Institute of Mental Health, Bethesda, MD, USA) [51]. The dry mass was determined after the leaves had been oven-dried at 70°C for 48 h to a constant mass. Finally, the LMA (gm^-2^) was calculated as the ratio of leaf dry mass to leaf area, and these measurements were repeated three times for each line.

The main yield traits, including tiller number per plant, filled grains per panicle, kilo-grain weightiness (g) and plant height (cm) of the 14 transgenic lines and WT were investigated in the paddy field and laboratory. All of the calculations described above were performed three times for each line in every group, and the experimental operations were conducted on T_6_ generation.

### Measurements of WSC levels, Rubisco activity, and chlorophyll content and calculation of *J*_cmax_

After the gas exchange measurements were performed, the same flag leaves were used to measure water soluble carbohydrates (WSCs: the sum of the concentrations of sucrose, glucose and fructose), Rubisco activity and chlorophyll content. The concentrations of WSCs in the extracts were measured using the modified anthrone procedure [52,53]. Rubisco activity was determined using a plant RUBPCase/Rubisco assay kit (GENMED SCIENTIFICS INC. USA), and the experimental protocol was conducted following the manufacturer’s instructions. The chlorophyll content of the leaf discs was determined by adapting the modified procedure described by Porra et al. [54]. Photographs were obtained to measure the leaf surface area, and the area values were calculated using the ImageJ software to convert mg/gFW to g/m^2^. Next, samples (0.2 g) of fresh leaf tissue were homogenized in 100% acetone at 4°C away from sunlight, and the fluorescence was measured at 663 nm and 645 nm with a spectrophotometer (TECAN, INFINITE M200 PRO) after centrifugation of the homogenates. The total chlorophyll content (mg/gFW) of the leaves was determined according to the modified equation described by Arnon [55]:

$$\left(8.04{\text{A}}_{663}+20.29{\text{A}}_{645}\right)\times \frac{\text{v}}{1000\text{w}}$$

The light-saturated potential rate of electron transport (*J*_max_) can be calculated according to the electron transport rate dependence on the chlorophyll concentration and was found to be 467 μmol photons (g Chl)^-1^s^-1^. The RuBP regenerative capacity (*J*_cmax_) was determined from *J*_max_ according to the equation of Li et al. [37] and Farquhar et al. [56]:

$$J_{\text{cmax}}=J_{\;\text{max}}/\left(4+4\Phi \right)$$

where Φ is the ratio of the oxygenation rate (*V*_o_) to the carboxylation rate (*V*_c_), which was assumed to have a constant value of 0.25. All of the experiments and calculations above were performed at least three times for each line.

### Microscopy

The midrib veins were removed from expanded flag leaf blades and cut into sections of approximately 5×2 mm. The sections were fixed and dehydrated following the protocol detailed by Scafaro et al. [57]. Transverse sections (thickness of 7 μm and 80 nm) were cut for light and transmission electron microscopy, respectively, and measured using ImageJ software. *S*_mes_ indicating the surface area of the mesophyll cells to the intercellular airspace was calculated as follows:

$$S_{mes}=F\frac{L_{mes}}{W}$$

where *F* is the curvature correction factor and is assumed to be 1.55 [57–59]. *L*_mes_ is the length of the mesophyll cells exposed to the intercellular airspace (μm), and *W* is the width of the analyzed section (μm). Leaf and mesophyll thickness were measured at the mid-point between vascular bundles and bulliform cells. All of the procedures described above were performed according to the methods reported by Scafaro et al. [57].

### Electron microscopy

Leaf samples for transmission electron microscopy were examined using a Tecnai G^2^ 20 S-TWIN transmission electron microscope (TEM). The percentage of the cell periphery adjacent to the IAS that was covered by chloroplasts and stromules (*S*_c_/*S*_mes_) was calculated as the length of the cell periphery facing the IAS covered by chloroplasts (*S*_c_) divided by the total length of the cell periphery facing the IAS (*S*_mes_). *S*_c_ was the surface area of the chloroplasts exposed to the intercellular airspace determined as follows:

$$S_{\text{c}}=\frac{L_{\text{c}}}{{L_{\text{mes}}}^{'}}{S_{\text{mes}}}^{'}$$

where *L*_mes_^'^ is the length of the mesophyll exposed to the intercellular airspace, and *L*_c_ is the length of the chloroplasts exposed to the intercellular airspace. The mesophyll cell wall thickness was measured and randomly selected from 6–12 sections per electron micrograph using ImageJ software, and these experimental manipulations were based on the instructions illustrated by Scafaro et al. [57].

### Measurements of stomatal density, stomatal size and stomatal index

To measure the stomatal density, stomatal index and stomatal size, the abaxial surface of the flag leaf was sampled separately from the WT and 14 transgenic lines at the flowering stage. These samples were first washed in sterile water and then fixed in FAA fixative (10% formaldehyde, 5% glacial acetic acid, 50% absolute ethanol, 35% sterile water). The samples were then washed serially in 50, 60, 70, 80, 90, 95 and 100% ethanol solutions, and the subsequent protocols were conducted according to the procedure for improved desiliconization and scraping described by Chen et al. [60]. All of the samples were viewed using DIC optics with a Nikon ECLIPSE 80i microscope equipped with a CCD camera, and the stomata and epidermal cells were counted in five square areas of 0.068 mm^2^ per leaf. The stomatal index (SI) was calculated using the following equation: SI = number of stomata / (number of stomatal + number of non-stomatal epidermal cells) × 100%. Stomatal and epidermal cell counts were determined for three repeats per plant from three individual plants of each line in every group.

### Statistical analysis

ANOVA (one-way analysis of variance) was applied to assess the differences for each parameter among transgenic and wild-type groups. Differences among means were established using Duncan’s test (P < 0.05). The data were analyzed applying the SPSS 10.0 program for Windows.

## Results

### Production and selection of rice transformants

The western blot analysis resulted in the identification of the target proteins ICTB and FBP/SBPase at 51.6 and 37.2 KD, respectively (Fig 1B), which were confirmed by southern blot analysis and RT-PCR (Fig 1C and 1D), and the constructs had inserted in the genomes of transgenic lines with only one copy of each gene. The results indicated that lines 40, 50, 51, 67 and 70 expressed *Ictb* (ICTB group), while lines 33, 53 and 80 expressed *FBP/Sbpase* (hereafter referred to as FS group), 41, 49 and 69 were *Ictb* and *FBP/Sbpase* expressing lines belonging to ICTB+FS group, lines 52, 61 and 81 were the empty construct group, and the WT group was 9311. The analyses of photosynthetic performance, mesophyll conductance to CO_2_ and other physiological traits were detected on the lines of these five groups.

### *In vivo* subcellular localization of ICTB and FBP/SBPase

**Fig 2:**
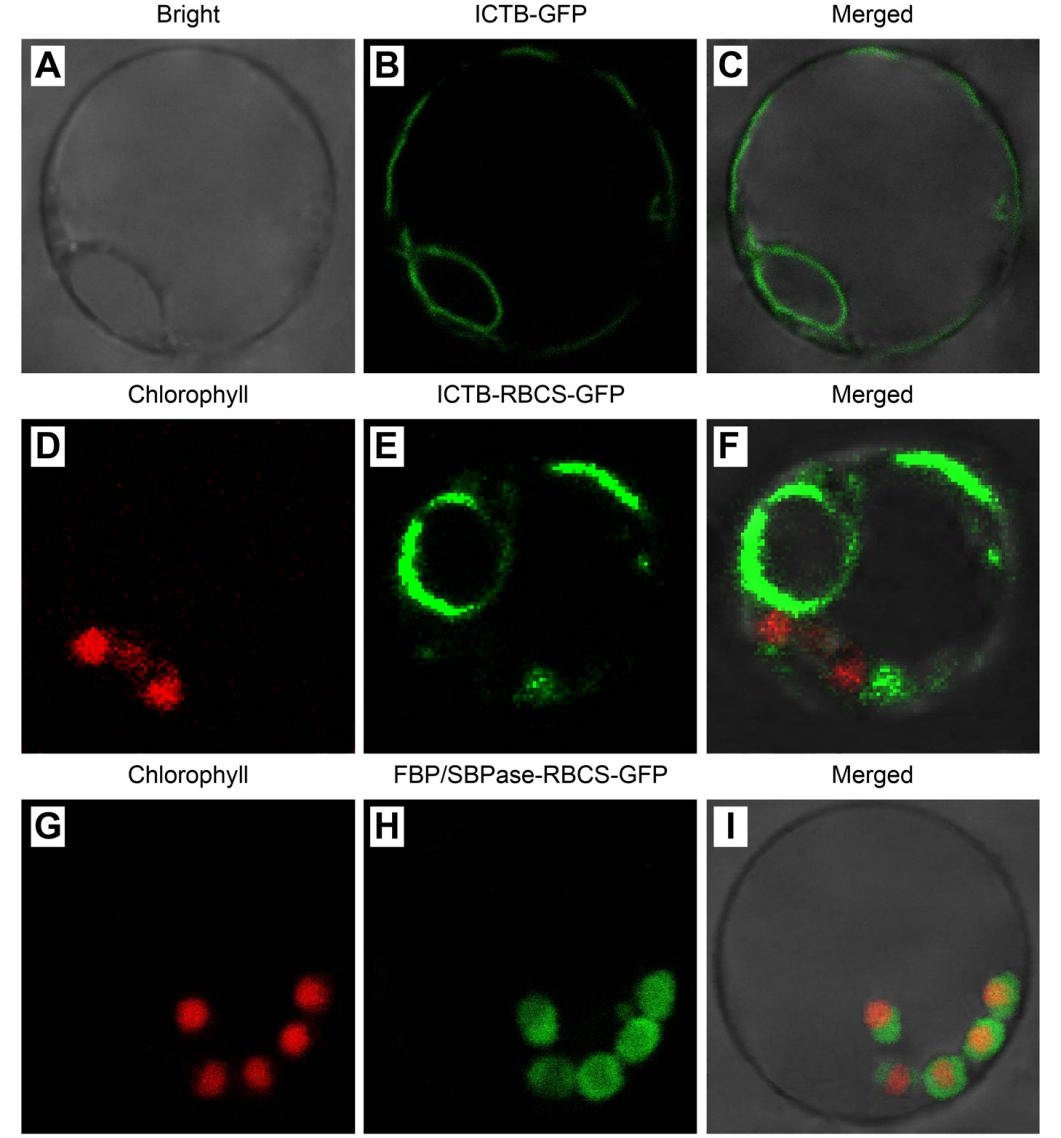
Subcellular localization of ICTB and FBP/SBPase protein. **A**, Bright field view of protoplasts. **B**, GFP signals from the ICTB-GFP fusion protein. **C**, Merged images. **D**, Red chlorophyll autofluorescence used as a chloroplast marker. **E**, GFP signals from the ICTB-RBCS-GFP fusion protein. **F**, Merged images. **G**, Red chlorophyll autofluorescence. **H**, GFP signals from the FBP/SBPase-RBCS-GFP fusion protein. **I**, Merged images, with yellow fluorescence in the merged images due to red chloroplast autofluorescence.

Successful transformation and expression of *Ictb* and *FBP/Sbpase* were achieved in rice, but it was unclear whether the proteins were localized in functional, interacting subcellular spaces related to CO_2_ transport and fixation. Consequently, the *in vivo* subcellular localization of ICTB and FBP/SBPase were tested by transiently expressing their fused GFP proteins in living rice protoplasts (Fig 2). The results showed that ICTB was present in the cytoplasm with or without RBCS signal peptide. In the present case, it was possible that our initial procedure was insufficient to deliver ICTB into chloroplasts and that the final destination was in the cytoplasm. Because eukaryotic cells are highly compartmentalized and the subcellular localization of a protein is intrinsic to its function [61]. The protein localization findings may support role for ICTB in transcellular carbon delivery to chloroplasts, as reported for aquaporins [62], which has a positive role in accelerating carbon diffusion and improving *g*_m_ in the liquid phase (Table 1). FBP/SBPase localized in the chloroplast and might promote CO_2_ assimilation in the transgenic lines based on our results (Table 1). These results meant that they exerted functions at different sites in the route of CO_2_ transport and assimilation respectively, thereby improving the photosynthetic efficiency.

**Table 1:** Photosynthetic parameters of the transgenic, WT and empty construct groups.

	Empty construct	ICTB	FS	ICTB+FS	WT
***g***_**m**_ **(mol CO**_**2**_ **m** ^**-2**^ **s** ^**-1**^ **)**	0.18 ± 0.04	0.21 ± 0.04*	0.24 ± 0.05* ☆	0.26 ± 0.05* ☆	0.19 ± 0.03
***P***_**n**_ **(μmol CO**_**2**_ **m** ^**-2**^ **s** ^**-1**^ **)**	13.1 ± 0.1	15.1 ± 0.6*	16.5 ± 0.1* ☆	17.9 ± 0.2* ☆	13.3 ± 0.1
**CE**	0.183 ± 0.023	0.177 ± 0.043	0.164 ± 0.011	0.181 ± 0.029	0.191 ± 0.073
***g***_**s**_ **(mol CO**_**2**_ **m** ^**-2**^ **s** ^**-1**^ **)**	0.16 ± 0.01	0.20 ± 0.01*	0.23 ± 0.01* ☆	0.27 ± 0.04* ☆	0.15 ± 0.01
**Rubisco activity (μmol CO**_**2**_ **min** ^**-1**^ **)**	0.62 ± 0.04	0.74 ± 0.02*	0.81 ± 0.03* ☆	0.87 ± 0.03* ☆	0.63 ± 0.03
**Chlorophyll content (g/m** ^**2**^ **)**	0.33 ± 0.03	0.36 ± 0.02	0.35 ± 0.02	0.34 ± 0.01	0.32 ± 0.02
***J***_**max**_ **(μmol photons m** ^**-2**^ **s** ^**-1**^ **)**	153.6 ± 14.9	169.2 ± 10.5	165.5 ± 9.6	157.2 ± 4.8	149.4 ± 9.3
***J***_**cmax**_ **(μmol CO**_**2**_ **m** ^**-2**^ **s** ^**-1**^ **)**	30.7 ± 3.0	33.8 ± 2.1	33.1 ± 1.9	31.4 ± 1.0	30.0 ± 1.9
**WSC (g/100 g)**	2.5 ± 0.1	3.2 ± 0.1*	3.6 ± 0.2* ☆	3.8 ± 0.1* ☆	2.6 ± 0.3
**LMA (gm** ^**-2**^ **)**	50.1 ± 0.3	52.8 ± 1.4*	56.6 ± 0.4* ☆	59.7 ± 0.8* ☆	50.3 ± 1.1

*Asterisks indicate a significant difference (P<0.05) from the wild type and empty construct.

☆Asterisks above ICTB+FS column indicate a significant difference (P<0.05) from two one-gene transgenic groups respectively.

☆Asterisks above FS column indicate a significant difference (P<0.05) from ICTB group.

Values are means ± SD for all lines with three biological replicates per group.

### Effect of *Ictb* and *FBPase/SBPase* on the variation in leaf anatomical properties

**Fig 3:**
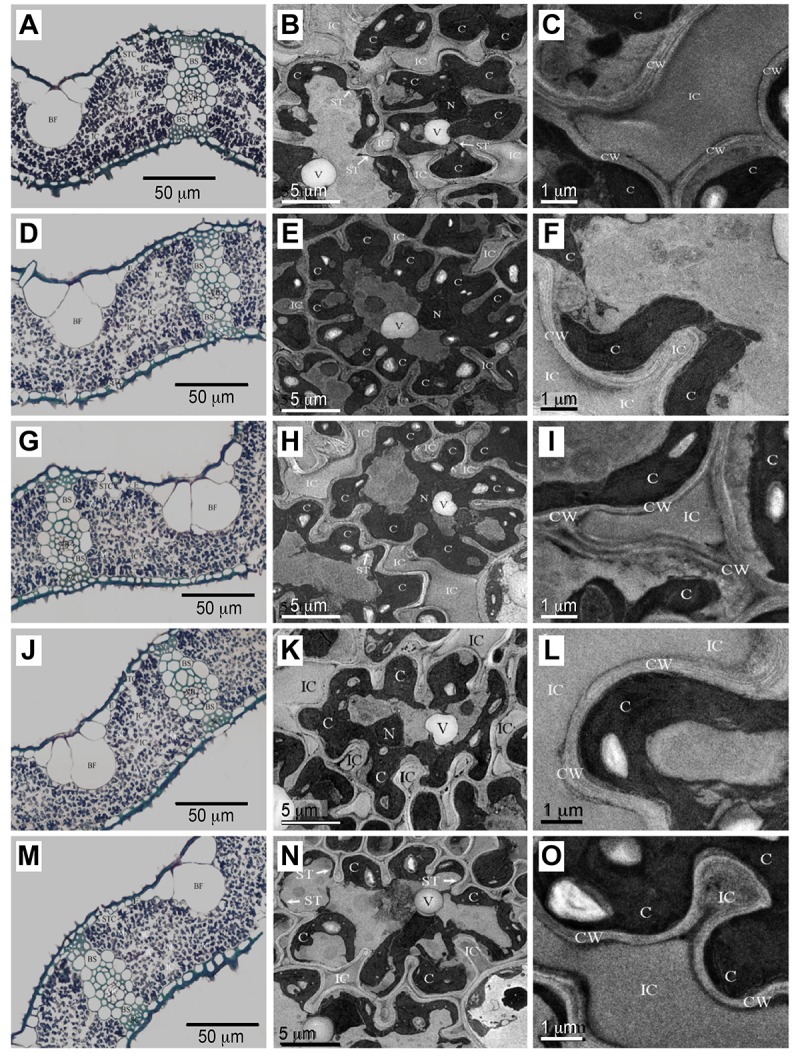
Light and transmission electron micrographs of transverse leaf sections from empty, transgenic and WT groups. Empty (A, B, C), ICTB (D, E, F), FS (G, H, I), ICTB+FS (J, K, L) and WT (M, N, O) lines. BF, bulliform cell; BS, outer bundle-sheath cell; C, chloroplast; CW, mesophyll cell wall; E, epidermis; IC, intercellular airspace; M, mesophyll cell; N, nucleus; SC, sclerenchyma strand; ST, stromule, indicated by white arrows; STC, stomatal cavity; V, vacuole; VB, vascular bundle.

We assessed whether the increased *g*_m_ and *P*_n_ in the transgenic groups resulted from the changes in leaf anatomy properties because leaf anatomy plays a major role in determining the mesophyll diffusion conductance to CO_2_ and, consequently, the variability in photosynthetic capacity among species [63]. Transverse sections of the leaf lamina examined by light and transmission electron microscopy revealed no significant leaf anatomical differences between the transgenic groups and WT (Fig 3, S1 Table). The five groups displayed similar leaf thicknesses, with numerical values of 89.8±4.9 μm, 90.6±3.5 μm, 91.5±3.6 μm, 91.3±4.2 μm and 90.8±6.1 μm for the empty construct, ICTB, FS, ICTB+FS and WT, respectively (S1 Table). The same trend was observed for the mesophyll wall thickness (Fig 3C, 3F, 3I, 3L and 3O, S1 Table). In rice, most of the cell periphery adjacent to the IAS is covered by chloroplasts and stromules. Furthermore, chloroplasts encompass almost the entire periphery of highly lobed cells, with stromules extend along the cell periphery that is not covered by chloroplasts (*S*_c_/*S*_mes_≧0.92; S1 Table). The values obtained for *S*_mes_ and *S*_c_ were not significantly different between the transgenic groups and WT (S1 Table), which is consistent with a previous report [64]. There were no differences in the number of chloroplasts per mesophyll area and the sizes of the chloroplasts, which were determining factors for *g*_m_. Similar results were obtained in the area occupied by epidermal or bulliform cells. All of the groups had the same amount of sclerenchymatous and bundle-sheath tissue, which may be an indicator of similar structural support. The cross-sectional area occupied by the vascular bundle and intercellular airspace was also similar between the transgenic groups and WT. Taken together, our results demonstrated that all of the transgenic groups and WT had a similar leaf anatomical structure (Fig 3), and the introduced target genes did not change the anatomical properties of the leaves. Moreover, these results further demonstrated that the transgenic groups and WT had similar inter-cellular and intracellular spaces and structures for CO_2_ diffusion and that the increased *g*_m_ and *P*_n_ in the transgenic groups was not due to the properties of the leaf anatomy.

### Lack of significant phenotypic variations in the stomatal traits of the transgenic groups compared to WT

The stomatal density and stomatal index of the three transgenic groups and WT at the flowering stages were shown in S1 Fig. The average stomatal density (SD) of the transgenic groups ranged from 746 to 756 mm^-2^ in comparison to 751 mm^-2^ for WT at the flowering stage. The average pavement cell (PC) density of the transgenic groups ranged from 1124 to 1153 mm^-2^, while the value obtained for WT was 1126 mm^-2^. The total cells (TCs) in the transgenic groups ranged from 1871 to 1909 mm^-2^ compared to 1877 mm^-2^ in WT (S1A Fig), and stomatal index (SI) in the three transgenic groups ranged from 0.39–0.40 compared with 0.40 in WT (S1B Fig). The results revealed that the two target genes did not substantially modify the stomatal distribution, which is the first component in the CO_2_ transport path, and these groups had almost the same stomatal structural properties and density, which showed that the improved photosynthetic performance was not due to the structural properties of the stomata.

### Impact of *Ictb* and *FBP/Sbpase* expression on photosynthetic performance in the flowering stages

**Fig 4:**
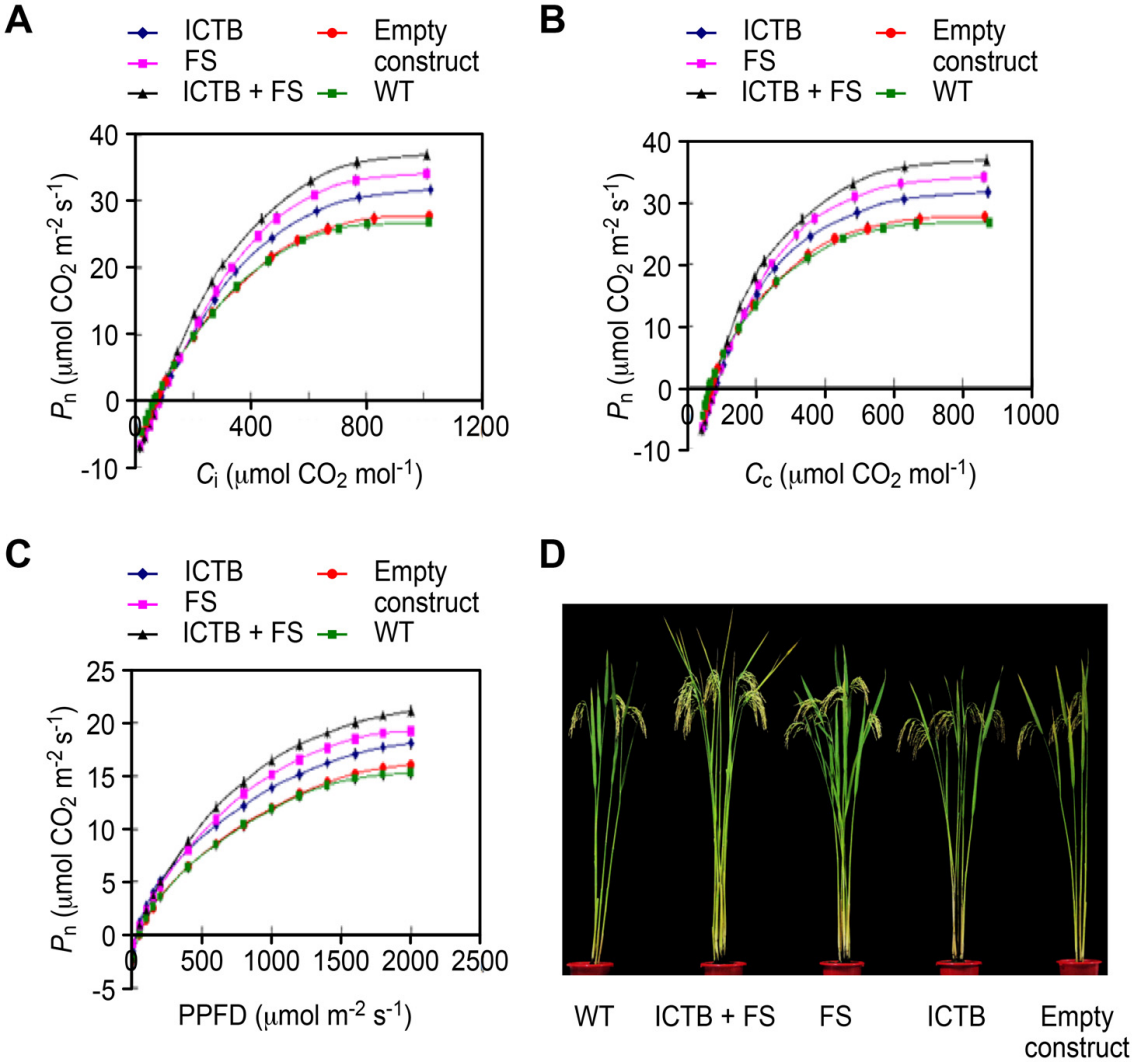
Differences in photosynthetic parameters measured using expanded flag leaves of rice plants between transgenic, WT and empty construct groups at the flowering stage. **A, B, C.** *P*_n_ responses to *C*_i_, *C*_c_ and PPFD respectively. Values are means ± SD for all lines with three biological replicates per group. **D.** Phenotype of wild-type, empty construct and three transgenic groups at the maturation stage.

Gas exchange measurements were used to investigate whether the *P*_n_ of three transgenic groups were higher than that of wild-type under the same light intensity and *C*_a_ in natural paddy fields in which the plants were grown. The response curve of *P*_n_ to varying *C*_i_ was assessed by measuring the CO_2_ uptake, which indicated that the difference became significant between transgenic groups and WT as the *C*_i_ increased above 200 μmol CO_2_ mol^-1^, the values of *P*_n_ tended to stabilize and attain saturation in all of the groups when the *C*_i_ levels exceeded 400 μmol CO_2_ mol^-1^ (Fig 4A). The changing range of *P*_n_ in the ICTB, FS, ICTB+FS groups was 31.3–31.9 (mean value 31.6±0.2), 33.4–34.5 (mean value 34.1±0.4) and 35.4–37.3 μmol CO_2_ m^-2^s^-1^ (mean value 36.8±0.6), respectively, while that of WT and the empty construct was 26.6–26.8 (mean value 26.7±0.1) and 27.3–28.3 (mean value 27.7±0.3) at the CO_2_ saturation points, respectively (data not shown). This result indicated that the *P*_n_ of three transgenic groups was higher than that of WT with values of approximately 18.4%, 27.7% and 37.8% respectively, and also suggested that the increased ability to perform a maximal photosynthetic rate in the transgenic groups was due to the functions of these two genes because the WT and empty construct groups displayed almost the same performance, and the photosynthetic ability increased in the order of *Ictb*, *FBP/Sbpase* and *Ictb+FBP/Sbpase* expression, which showed greater improvements in the photosynthetic capacity with *FBP/Sbpase* than *Ictb*. An additive effect was observed in the ICTB+FS groups (*Ictb+FBP/Sbpase*).

Obtaining an estimate of *g*_m_ allows the simple conversion of *P*_n_-*C*_i_ curves into response curves of *P*_n_ to *C*_c_ [65]. Using this method, we found that differences in the curves between the three transgenic groups and WT became significant and stabilized when *C*_c_ was higher than 400 μmol CO_2_ mol^-1^ roughly (Fig 4B), and this trend was identical to that of the *P*_n_/*C*_i_ response curve. Taken together, these results indicated that the two target genes can improve the CO_2_ assimilation capacity of the transgenic groups compared with WT and empty construct groups based on the same *C*_i_ or *C*_c_.

The response of *P*_n_ to PPFD was measured (Fig 4C) under the same atmospheric conditions (*C*_a_ = 380 ± 10 μmolmol^-1^; RH = 80 ± 5%) but with various light intensities (0–2,000 μmol m^-2^s^-1^) at 35°C. At irradiances below 200 μmol m^-2^s^-1^, the three transgenic groups did not display clear difference in photosynthetic rates with respect to WT. As the light intensity increased, the photosynthetic rates of the three transgenic groups increased significantly compared to WT when the light intensity ranged from 500 to 2,000 μmolm^-2^s^-1^. Photo-saturation of photosynthesis was observed when the light intensity exceeded 1,500 μmol m^-2^s^-1^. In comparison to WT, the light saturation point for the transgenic groups ICTB, FS and ICTB+FS was higher by approximately 17.9%, 25.5% and 37.5%, respectively, and the value obtained for the empty construct was very similar to that of WT. The results indicated that the light conversion efficiency increased sequentially in the three transgenic groups and exhibited an additive effect in the ICTB+FS group. This result could be explained by the two genes having the ability to promote CO_2_ flux and *P*_n_ and further increase the demands for light energy in rice.

### The related photosynthetic parameters exhibited a significant increase in transgenic groups compared to WT

The *P*_n_-PPFD and *P*_n_-*C*_i_ curves in Fig 4 illustrated significant differences in *P*_n_ at a PPFD of 1200 μmol m^-2^s^-1^ and *C*_a_ of 380 μmol CO_2_ mol^-1^ among the different groups. In three transgenic groups, ICTB+FS displayed the highest values (17.9 μmol CO_2_ m^-2^s^1^), ICTB had the lowest values (15.1), and FS displayed values in the middle (16.5), which were 36.6%, 15.3% and 26.0% higher than the 13.1 of WT, respectively (Table 1). In addition, the *P*_n_ value for ICTB+FS was higher than that of the ICTB and FS groups by 18.5 and 8.5%, respectively, and the FS value was significantly higher than that determined for the ICTB groups by 9.3%. Similar differences were observed in *g*_s_ (stomatal conductance to CO_2_) and *g*_m_. Likewise, clear differences were observed in the total activity of Rubisco, LMA and WSC (Table 1), demonstrating the same trend as observed for *P*_n_ illustrated above.

*C*_c_ is the ultimate limiting factor for light-saturated photosynthesis after CO_2_ enters the stomatal pore. CO_2_ molecules must pass through other phases to reach the carboxylation site (*C*_c_), i.e., the cell wall, plasmalemma, cytosol, chloroplast envelope and stroma. Many recent studies have demonstrated that *P*_n_ depends primarily on the transportation of CO_2_ [45]. To estimate *g*_m_ and CE under conditions of PPFD of 1200 μmol m^-2^s^-1^, 35°C and *C*_a_ = 380 μmol CO_2_ mol^-1^, measurements of gas exchange and chlorophyll fluorescence were conducted and analyzed concurrently [65,66]. Our results showed that the values for *g*_m_ in the three transgenic groups ICTB, FS and ICTB+FS, were 10.5%, 26.3% and 36.8% higher than WT, respectively. Nevertheless, the corresponding values for CE didn’t show significant increment since there were no differences of *P*_n_ values between groups as *C*_c_ was <200 μmol CO_2_ mol^-1^ indicated by the *P*_n_/*C*_c_ curves (Table 1, Fig 4B). These results demonstrated that the efficiency of CO_2_ transport from the air to the carboxylation site had been improved and further increased *P*_n_ based on the growth trend observed for the ICTB, FS and ICTB+FS groups. The results also indicated that the cyanobacterial *Ictb* and *FBP/Sbpase* genes promoted CO_2_ transport capacity and carbon fixation efficiency in rice.

Compared to WT, similar chlorophyll content were observed in the leaves of the three transgenic groups, resulting in almost the same value for *J*_max_ (Table 1). In our study, 2,000 μmol photons m^-2^s^-1^ was a saturating light intensity due to the stable value of *P*_n_ obtained for these groups at the atmospheric CO_2_ concentration, and it could provide sufficient ATP, NADPH, and RuBP for carboxylation that was significantly less than *J*_cmax_ (Fig 4C; Table 1). These results suggested that the RuBP regeneration rate was not a limiting factor for the light-saturated photosynthetic rate. With the increase in *C*_c_, *P*_n_ increased to approach the value of *J*_cmax_, and *C*_c_ was >600 μmol CO_2_ mol^-1^ in all of the groups when *P*_n_ attained the maximal value (Fig 4B). All of the above findings indicated that *C*_c_ was the ultimate limiting factor for photosynthesis when *C*_c_ was not saturated [37].

### Yield traits and plant height were partly improved by *Ictb* and *FBP/Sbpase*

The rice grain yield is mainly determined by three components, including the tiller number per plant, filled grain number per panicle, and the grain weight [67]. In the present study, only the tiller number per plant in the ICTB+FS group displayed a significant increment compared with FS, ICTB and WT group respectively, while the other two components did not show an increase (S2A–S2C Fig). In FS group, kilo-grain weightiness decreased significantly compared to WT and ICTB group respectively although tiller number and filled grains per panicle were slightly higher than that of WT. As for ICTB group, the three components did not exhibit any increase in contrast with WT, and it seemed the main yield traits in the one-gene transgenic groups had not been improved. In addition, the plant height in three transgenic groups were clearly increased compared with the WT plants, and ICTB group showed significant while ICTB+FS and FS group showed great significant increment (Fig 4D; S2D Fig). The results indicated that the tillering capacity in ICTB+FS and the biomass above ground of three transgenic groups were significantly higher than those of WT.

## Discussion

### Leaf anatomy and mesophyll conductance to CO_2_

The photosynthetic rate in plants is determined by the velocity of the diffusion of CO_2_ through stomata and the capacity to fix CO_2_ into sugar. The stomatal pore and the path through the mesophyll from the cell wall to Rubisco are two primary resistance to CO_2_ diffusion. The mesophyll pathway comprises a series of physical barriers to CO_2_ diffusion, including cell walls, lipid membranes, liquid cytoplasm and stroma [63,68]. Our basic goal was to reduce the resistance in the mesophyll pathway by introducing the cyanobacterial *Ictb* gene to boost mesophyll conductance, which is also influenced by the structure of the leaf [49,57,63,69–71]. The ultimate goal was to increase the CO_2_ carboxylation efficiency by introducing another cyanobacterial *FBP/Sbpase* gene in rice. However, our results showed that the leaf anatomy was similar in almost all respects between the three transgenic groups, WT and the empty construct, and there were no differences in the stomatal density, *S*_c_/*S*_mes_, thickness of the mesophyll cell wall or chloroplast size, which are critical structural components of a leaf that affect *g*_m_ [37,63,68]. This finding indicated that the increased *g*_m_ and *P*_n_ in the transgenic groups was not due to the effect of the leaf anatomy properties and mesophyll cell structure but to the biochemical functions of ICTB and FBP/SBPase. In addition, the expression of *Ictb* and *FBP/Sbpase*, whether alone or together, would modify the diffusion limitations along the biochemical path of CO_2_ transport rather than the physical diffusion conductance inside the leaves.

Many previous analyses have demonstrated that *g*_m_ and *P*_n_ change to permit adaptation to the environmental conditions in which the species evolves, such as light, nutrients, water and temperature [37,72–76]. In our study, the transgenic groups and WT were planted in the same paddy field with the same ecological niche, and thus, different effects of environmental factors on leaf and mesophyll cell structure and properties between transgenic groups and WT were precluded. The present results indicated that the two target genes were not sufficient to modify the leaf anatomy structure, such as the arrangement of mesophyll cells in the CO_2_ diffusion path and the surrounding chloroplasts directly abutting the mesophyll cells. The results also suggested that the increased *P*_n_ and *g*_m_ were not related to the leaf anatomy structure but were due to the biochemical functions of the two target genes. In addition, the results indirectly proved that the leaf anatomy structure was determined mainly by the environmental conditions, as suggested by previous studies such as Assuero et al. [77] and Gomez-Del-Campo et al. [78].

Flexas et al. [31] demonstrated the occurrence of an evolutionary trend towards a higher *g*_m_, and the photosynthetic capacity of angiosperms was greatly increased following the cretaceous period in association with the changes in leaf morphology. Evolutionary pressure resulted in the evolution of strategies to efficiently acquire inorganic carbon for photosynthesis [79]. In addition, cultivated rice has been domesticated and selected for approximately 8000–10,000 years [80], and it shows extensive stromule formation that allows *S*_c_/*S*_m_ to approach 1.0, which is consistent with our findings and higher than the values observed for species without stromules in which *S*_c_/*S*_m_ never exceeds 0.8 [81]. These observations support the hypothesis that stromules in photosynthetic cells function to seal breaches between adjacent chloroplasts, thus improving the trapping mechanism of photorespired and respired CO_2_ [59,81]. However, the adjustment in mesophyll cell thickness, geometry and packing according to ambient light conditions may function to control the distribution of internal light and maximize light absorption and carbon fixation within the leaf [82]. Considered together with our results, these observations demonstrated that rice had evolved a sophisticated leaf anatomy structure and cellular ultrastructure for trapping CO_2_ from the intercellular airspace and created optimal conditions for improving the photosynthetic rate. In the present study, the two target genes were related to CO_2_ transport and fixation but not to the cell structure, and it seems plausible that it was very difficult to change the mesophyll structure under natural environmental conditions.

### The effect of *Ictb* and *FBP/Sbpase* on biochemical functions, physiological and yield traits

In our results, Rubisco activity of transgenic groups increased significantly compared with WT and the empty construct. The values for CE showed no differences as *C*_c_ was <200 μmol CO_2_ mol^-1^, and *P*_n_ exhibited marked difference along with the increasing of *C*_c_ when *C*_c_ was >200 μmol CO_2_ mol^-1^. These results supported the previous conclusions that the carboxylation capacity and activity of Rubisco are regulated by the chloroplastic CO_2_ concentration and which is also generally considered to be an ultimate limiting factor for CO_2_ fixation [37]. Thus, ICTB and FBP/SBPase could promote carbon diffusion and stimulated the activity and carboxylation capacity of Rubisco alone or together and then achieved higher *P*_n_ at CO_2_ saturation point. The other photosynthetic parameters determined for the empty construct group were very similar to those of WT, which indicated that the expression construct alone without target genes could not change the photosynthetic capacity and that the increased parameters in the transgenic groups was not a consequence of the construct sequence or a mutation in the host genome due to an insertion. Thus, in the ICTB+FS group, the functional additive effect could be explained by the ability of ICTB to boost the carbon flux and drive more CO_2_ into the chloroplast. Then, the Calvin cycle is accelerated by *FBP/Sbpase* to consume and convert carbon to avoid carbon redundancy. Carbon transport and carboxylation reactions were linked together by these two genes and improved simultaneously, and they were usually associated with the photosynthetic capacity.

The expression of *Ictb* and *FBP/Sbpase* also exerted positive effects on LMA and WSC compared with WT, but no differences were observed between them in terms of the anatomical structure of the leaf. A tentative conclusion might be drawn from these results as follows, the increased LMA primarily resulted from the increased WSC, and the increased WSC produced by the improved photosynthesis was not used to build the structural components such as support tissues and cell wall, which involve stronger CO_2_ diffusion limitations for photosynthesis and likely only accumulated chemical substances in mesophyll cells. In the three transgenic groups, ICTB+FS exhibited the highest value for LMA and WSC (Table 1) and showed higher tiller number (S2A Fig). These results indicated that the capacity to preserve and transport WSC to grains increased only under the addition effect of ICTB and FS, although the photosynthetic parameters in FS and ICTB groups showed significant increment compared to that of WT. These results inferred that photosynthetic capacity was not always related to the yield traits positively, and the potential underlying mechanisms require further analysis because rice yield is considered to be a quantitative trait that is controlled by multiple genes [83]. These findings also demonstrated that neither expression of both nor one of the target genes was sufficient to improve all of the yield traits, and potential problems associated with improving the rice yield in the transgenic groups might remain unresolved due to the limitations of our techniques. The most probable explanation was that the carbohydrate export pathway from source to sink should be facilitated to avoid carbohydrate redundancy in mesophyll cells.

### The relationships of *Ictb*, *FBP/Sbpase* and light energy conversion capacity

In addition to the velocity of CO_2_ diffusion through stomata and the capacity to fix CO_2_ into sugar, photosynthesis in plants has also been considered for decades to be limited by the capacity of the photosynthetic machinery to convert light energy into biochemical energy. Our results revealed no differences in chlorophyll content among the three transgenic groups compared with WT and the empty construct group, which had similar values for *J*_max_ and *J*_cmax_. Although the *Ictb* and *FBP/Sbpase* genes were not directly related to light absorbance and conversion, they promoted *g*_m_ and the light-saturated photosynthetic rate compared with WT. Considering that *J*_max_ is maximal light-driven electron flux and can be used to estimate the potential photosynthetic capacity derived from light and the capacity for RuBP regeneration, these results indicated that the capacity of the photosynthetic machinery to convert light into biochemical energy and the RuBP regenerative capacity were similar and sufficient to increase *P*_n_ and *g*_m_. A possible explanation was that these two increased photosynthetic parameters was not related to the capacity to convert light energy but mainly to the biochemical function of these two proteins alone or combined.

### The functional synergistic effect of *Ictb* and *FBP/Sbpase*

Subcellular localization studies suggested that FBP/SBPase was present in chloroplasts and ICTB was in the cytoplasm with or without RBCS signal peptide. These studies indicated that our initial transgenic procedures could not deliver ICTB into chloroplasts, and the potential observed mechanisms were likely a result of an eclipsed distribution [61,84]. These findings could be ascribed to its intrinsic function as a membrane protein and a natural address in the cytoplasm, coinciding with the assertion that ICTB is related to the endoplasmic reticulum (ER) and can cause an accumulation of unfolded membrane proteins in the ER to produce a classic ER shock response, as proposed by Agarwal et al. [85], Urade [86] and Price et al. [13]. Nevertheless, the ICTB protein alone had a positive effect on photosynthetic parameters such as *g*_m_ and *P*_n_ despite its location outside the chloroplast, in accordance with our initial purpose, because it could deliver CO_2_ more effectively through the cytomembrane and liquid phase cytoplasm, which are considered important obstacles to carbon flux. These findings and the results of Simkin et al. [87] provided partial answers to the question proposed by Price et al. [13] and clues for further studies of the function of *Ictb*.

Based on the assertions illustrated above, a positive effect in two one-gene groups and an additional effect in the ICTB+FS group, the two target proteins likely have a synergistic interaction which was also confirmed in tobacco by Simkin et al. [87], and this interaction without a physical binding could be interpreted as a functional interaction according to Bassel et al. [88]. Thus, ICTB potentially decreased the CO_2_ transfer resistance in mesophyll cells, and FBP/SBPase promoted carbon assimilation in chloroplasts and improved the overall photosynthetic capacity in the transgenic groups.

### Conclusions and Expectations

The *g*_m_ is an important limiting factor in photosynthesis, comprising ~20 to ~50% of the photosynthetic limitations [89], and *C*_s_/*C*_a_ = 0.60 to 0.85 (*C*_s_, CO_2_ concentration in the sub-stomatal cavity). However, the amount of CO_2_ drawn from the sub-stomatal cavity to the bulk intercellular spaces is small, and the ratio is as follows: *C*_i_/*C*_s_ = 0.90 to 0.99 [68]. The two values can be converted to *C*_i_/*C*_a_ = 0.7, which is remarkably constant across C_3_ species [90]. Techniques to improve the velocity of CO_2_ absorbance from intercellular space into mesophyll cells (that is, how to utilize CO_2_ in the intercellular space sufficiently to increase *g*_m_) is an important research topic. Because it is impractical to change the structure of the stomatal cavity due to its bi-functionality and regulation of water vapor diffusion [91], an effective method is to improve the permeability of the cell wall and chloroplast envelope to CO_2_ transport because they have been identified as major limiting components to CO_2_ transport [68].

ICTB was located in the cytoplasm and contributed to improvements in *g*_m_ and *P*_n_, while FBP/SBPase was located in chloroplasts and demonstrated improved functionality compared with ICTB. These results inferred the function of the genes operating in the Calvin cycle (located in chloroplasts) were more important than those only acting on the carbon diffusion (located in cytoplasm) for the promotion of photosynthesis. This results explained the acceleration of the Calvin cycle to more efficiently consume and convert carbon, and both an intense requirement for CO_2_ and the promotion of *g*_m_ and *P*_n_ were observed. These effects were more pronounced than those in ICTB group, although ICTB alone could also promote *g*_m_ and *P*_n_.

Based on the better performance of the yield traits in the ICTB+FS group and the ideal tactics illustrated above, we think that the most effective method is the transformation of a key gene in the Calvin cycle and other genes that function at different sites in the carbon flux pathway to form a “bead-like” pattern and improve the entire efficiency of CO_2_ transport and fixation. We are attempting to assemble a CO_2_ transport and assimilation (Calvin cycle) chain to improve the photosynthetic rate and yield traits via multigene transfer (MGT), and we anticipate that this issue will become a high priority in future studies.

## Supporting Information

S1 Fig Phenotypic distributions of the stomatal density at the flowering stage of the three transgenic, WT and empty construct groups grown in the same paddy field. (TIF)

S2 Fig Agronomic traits of the transgenic rice groups, WT and empty construct groups. (TIF)

S1 File The full length of *Ictb* and *FBP/Sbpase* CDS sequence. (DOC)

S1 Table Leaf anatomical properties of three transgenic, WT and empty construct groups. (DOC)
