# Supplementary figures and images for: Transgenic Rice Expressing Ictb and FBP/Sbpase Derived from Cyanobacteria Exhibits Enhanced Photosynthesis and Mesophyll Conductance to CO2
Source: PLoS One. 2015 Oct 21;10(10):e0140928. doi: 10.1371/journal.pone.0140928 (PMC4638112; doi:10.1371/journal.pone.0140928)

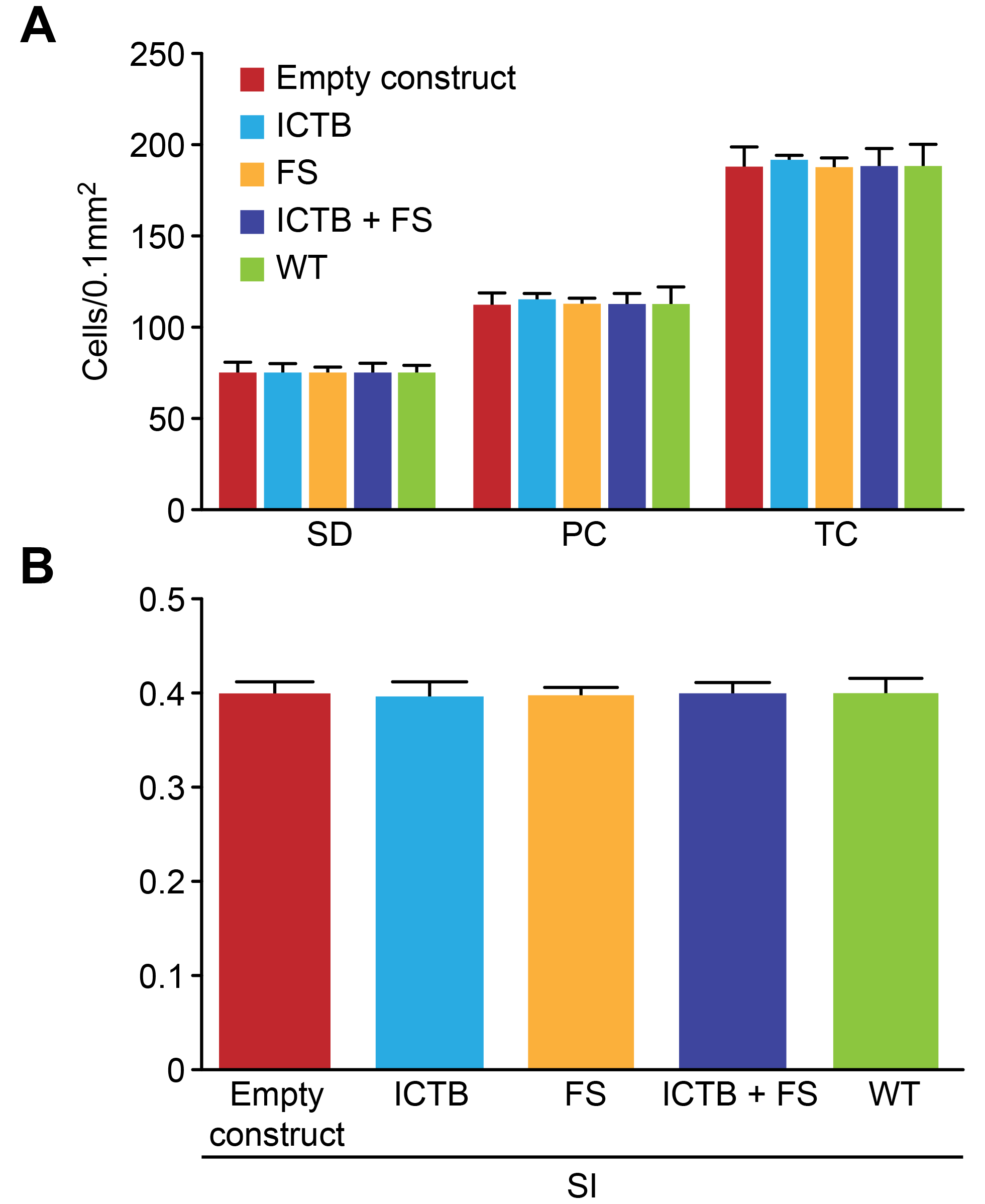

Supplement: S1 Fig — (TIF) [file pone.0140928.s001.tif]

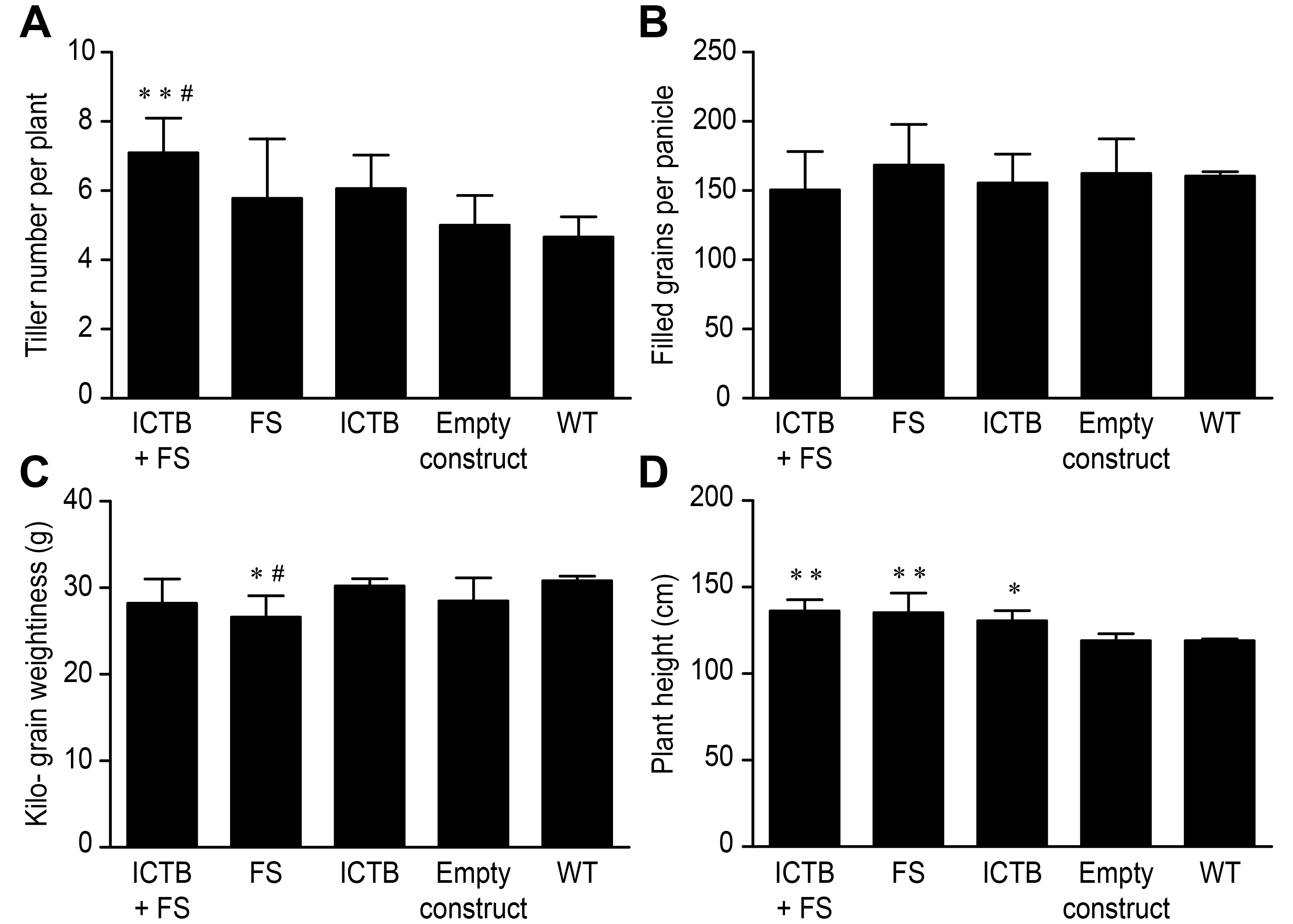

Supplement: S2 Fig — (TIF) [file pone.0140928.s002.tif]
