# Supplementary material for: Transgenic Rice Expressing Ictb and FBP/Sbpase Derived from Cyanobacteria Exhibits Enhanced Photosynthesis and Mesophyll Conductance to CO2
Source: PLoS One. 2015 Oct 21;10(10):e0140928. doi: 10.1371/journal.pone.0140928 (PMC4638112; doi:10.1371/journal.pone.0140928)
Supplement: S1 File — (DOC) [file pone.0140928.s003.doc]

### Transgenic Rice Expressing *Ictb* and *FBP/Sbpase* Derived from Cyanobacteria Exhibits Enhanced Photosynthesis and Mesophyll Conductance to CO_2_

Han Yu Gong^1, 2^,·Yang Li^1^, ·Gen Fang^1^,·Dao Heng Hu^1^,·Wen Bin Jin^1^,·Zhao Hai Wang^1^,·Yang Sheng Li^1^*

**Supplementary Data**

**Materials and Methods**

**S1 File. The full length of *Ictb* and *FBP/Sbpase* CDS sequence**

**1. *Ictb* (*dc14*) (GenBank Locus: SPU62616)**

atgactgtctg gcaaactctg acttttgccc attaccaacc ccaacagtgg ggccacagca gtttcttgca tcggctgttt ggcagcctgc gagcttggcg ggcctccagc cagctgttgg tttggtctga ggcactgggt ggcttcttgc ttgctgtcgt ctacggttcg gctccgtttg tgcccagttc cgccctaggg ttggggctag ccgcgatcgc ggcctattgg gccctgctct cgctgacaga tatcgatctg cggcaagcaa cccccattca ctggctggtg ctgctctact ggggcgtcga tgccctagca acgggactct cacccgtacg cgctgcagct ttagttgggc tagccaaact gacgctctac ctgttggttt ttgccctagc ggctcgggtt ctccgcaatc cccgtctgcg atcgctgctg ttctcggtcg tcgtgatcac atcgcttttt gtcagtgtct acggcctcaa ccaatggatc tacggcgttg

aagagctggc gacttgggtg gatcgcaact cggttgccga cttcacctca cgggtttaca gctatctggg caaccccaac ctgctggctg cttatctggt gccgacgact gccttttctg cagcagcgat cggggtgtgg cgcggctggc tccccaagct gctggcgatc gctgcgacag gtgcgagcag cttatgtctg atcctcacct acagtcgcgg tggctggctg ggttttgtcg ccatgatttt tgtctgggcg ttattagggc tctactggtt tcaaccccgt ctacccgcac cctggcgacg ctggctattc ccagtcgtat tgggtggact agtcgcggtg ctcttggtgg cggtgcttgg acttgagccg ttgcgcgtgc gcgtgttgag catctttgtg gggcgtgaag acagcagcaa caacttccgg atcaatgtct ggctggcggt gctgcagatg attcaagatc ggccttggct gggcatcggc cccggcaata ccgcctttaa cctggtttat cccctctatc aacaggcgcg ctttacggcg ttgagcgcct actccgtccc gctggaagtc gcggttgagg gcggactact gggcttgacg gccttcgctt ggctgctgct ggtcacggcg gtgacggcgg tgcggcaggt gagccgactg cggcgcgatc gcaatcccca agccttttgg ttgatggcta gcttggccgg tttggcagga atgctgggtc acggtctgtt tgataccgtg ctctatcgac cggaagccag tacgctctgg tggctctgta ttggagcgat cgcgagtttc tggcagcccc aaccttccaa gcaactccct ccagaagccg agcattcaga cgaaaaaatg tag

**2. *FBP/Sbpase* (GenBank Locus: D49680)**

atgg ctcaatccac cacttccgag acccacacca gagatctcga tcgggattgc accaccctgt cgcgccatgt tctcgaacag ttccaaagct ttagccccga agctcaagac cttgcggcgt tgatgcagcg cattggcctt gccgctaagc taatcgcgcg tcgcctcagc cacgcggggc tggtggatga tgccctcggt ttcacggggg aaatcaacgt ccagggcgaa gcgtcaaaac ggatggacgt ctacgctaac caagttttca tctcagtttt tcggcagagt ggcttggtct gtcggctggc ttccgaggaa atggagaagc cctactacat tcctgaaaat tgcccgatcg gtcgctatac gctgctctac gaccctttag atggctcggc caacgtcgat gtcgatctca acgtcggttc catttttgcg gtgcggcgtc aagagttcta cgacgagtcc catgaagcca aggatcttct gcagccaggc gatcgccaaa ttgctgcggg ctatgtcctc tatggggcca gcaccttgct ggtctacagc atgggtcagg gcgtccatgt ttttgtactg gaccccagct taggtgagtt tgtcctcgcg cagtccgaca tccagctccc gaactcgggt cagatctaca gcgtcaatga agggaacttt tgcagtggcc cgaaggtatc gccagtacat ccgggaaatg catcgccgcg aaggctacag cggcgctata gcggagcgtt ggtagctgac ttccatcgca ttttgatgca gggtggtgtg tttctctatc ccgaaaccgt caagaatccc actggcaagc tgcgactgct ctacgaagca ccaccgatgg cctttctggc tgaacaagca ggcggcaagg ccagtgacgg ccagaagcct atcctcttgc gccaacccca agccctgcac cggaggtgcc cgctaattat cggtagcgcc gcagatgtgg actttgtcga cagcctgctt ggctga

**S1 Fig. Phenotypic distributions of the stomatal density at the flowering stage of the three transgenic, WT and empty construct groups grown in the same paddy field.** **A.** Comparisons of stomatal density; **B.** SI: stomatal index. SD: stomatal density, PC: pavement cell, TC: total cell. Values are means ± SD of all lines in each group for three biological replicates.

**S2 Fig. Agronomic traits of the transgenic rice groups, WT and empty construct groups. A.** Tiller number per plant; **B.** Filled grains per panicle; **C.** Kilo-grain weightiness; **D.** Plant height

Significant differences between transgenic groups and WT were indicated by * (P < 0.05) and ** (P < 0.01).

**#** in A indicate significant difference between ICTB+FS and FS, ICTB group respectively (P < 0.05).

**#**  in C indicate significant difference between FS and ICTB group.

Values are means ± SD of all of the lines per group from T_6_ generations for three replicates.

S1 Table. Leaf anatomical properties of three transgenic, WT and empty construct groups

|  | **Empty construct** | **ICTB** | **FS** | **ICTB+FS** | **WT** |
| --- | --- | --- | --- | --- | --- |
| **Leaf thickness (μm)** | 89.8 ± 4.9 | 90.6 ± 3.5 | 91.5 ± 3.6 | 91.3 ± 4.2 | 90.8 ± 6.1 |
| **Mesophyll thickness (μm)** | 81.4 ± 4.7 | 80.6 ± 3.6 | 82.1 ± 4.4 | 81.8 ± 4.3 | 80.8 ± 3.5 |
| **Mesophyll cell wall thickness (nm)** | 169.7 ± 7.8 | 171.8 ± 3.6 | 170.3 ± 3.2 | 170.3 ± 3.4 | 168.8 ± 2.5 |
| ***S*_mes_ (m^2^m^-2^)** | 18.9 ± 0.3 | 17.1 ± 0.5 | 18.9 ± 0.7 | 17.1 ± 0.6 | 18.8 ± 0.8 |
| ***S*_c_ (m^2^m^-2^)** | 17.8 ± 0.3 | 17.7 ± 0.5 | 17.5 ± 0.4 | 17.5 ± 0.6 | 17.4 ± 0.7 |
| ***S*_c_/*S*_mes_** | 0.92 ± 0.03 | 0.93 ± 0.01 | 0.93 ± 0.02 | 0.92 ± 0.01 | 0.92 ± 0.02 |
| **Chloroplasts per m^-2^ mesophyll (10^9^)** | 65.7 ± 6.5 | 66.3 ± 6.3 | 66.4 ± 4.7 | 66.1 ± 5.8 | 65.9 ± 4.6 |
| **Chloroplast size (mm^2^)** | 10.2 ± 2.9 | 10.2 ± 3.4 | 10.3 ± 4.5 | 10.2 ± 3.5 | 10.3 ± 4.7 |
| **Intercellular airspace (% section)** | 13.3 ± 0.7 | 13.4 ± 0.4 | 13.5 ± 0.2 | 13.2 ± 0.3 | 13.3 ± 0.6 |
| **Mesophyll cells**  **(% section)** | 42.5 ± 1.4 | 42.8 ± 1.8 | 42.3 ± 3.8 | 42.4 ± 3.6 | 42.3 ± 2.6 |
| **Epidermis**  **(% section)** | 13.1 ± 1.1 | 13.1 ± 1.4 | 13.1 ± 1.3 | 13.2 ± 1.4 | 13.3 ± 1.7 |
| **Bulliform cells**  **(% section)** | 10.4 ± 0.5 | 10.3 ± 0.6 | 10.5 ± 0.4 | 10.5 ± 0.3 | 10.3 ± 0.2 |
| **Sclerenchyma strands (% section)** | 4.6 ± 0.1 | 4.5± 0.5 | 4.6 ± 0.2 | 4.6 ± 0.4 | 4.7 ± 0.3 |
| **Outer bundle-sheath (% section)** | 9.3 ± 0.7 | 9.3 ± 0.2 | 9.4 ± 0.1 | 9.5 ± 0.6 | 9.6 ± 0.3 |
| **Vascular bundle**  **(% section)** | 6.8 ± 0.5 | 6.6 ± 0.6 | 6.6 ± 0.3 | 6.6 ± 0.4 | 6.5 ± 0.6 |

Values are means ± SD of all lines for three biological replicates in every group.
