# Supplementary material for: Transgenic Rice Expressing Ictb and FBP/Sbpase Derived from Cyanobacteria Exhibits Enhanced Photosynthesis and Mesophyll Conductance to CO2
Source: PLoS One. 2015 Oct 21;10(10):e0140928. doi: 10.1371/journal.pone.0140928 (PMC4638112; doi:10.1371/journal.pone.0140928)
Supplement: S1 Table — (DOC) [file pone.0140928.s004.doc]

S1 Table. Leaf anatomical properties of three transgenic, WT and empty construct groups

|  | **Empty construct** | **ICTB** | **FS** | **ICTB+FS** | **WT** |
| --- | --- | --- | --- | --- | --- |
| **Leaf thickness (μm)** | 89.8 ± 4.9 | 90.6 ± 3.5 | 91.5 ± 3.6 | 91.3 ± 4.2 | 90.8 ± 6.1 |
| **Mesophyll thickness (μm)** | 81.4 ± 4.7 | 80.6 ± 3.6 | 82.1 ± 4.4 | 81.8 ± 4.3 | 80.8 ± 3.5 |
| **Mesophyll cell wall thickness (nm)** | 169.7 ± 7.8 | 171.8 ± 3.6 | 170.3 ± 3.2 | 170.3 ± 3.4 | 168.8 ± 2.5 |
| ***S*_mes_ (m^2^m^-2^)** | 18.9 ± 0.3 | 17.1 ± 0.5 | 18.9 ± 0.7 | 17.1 ± 0.6 | 18.8 ± 0.8 |
| ***S*_c_ (m^2^m^-2^)** | 17.8 ± 0.3 | 17.7 ± 0.5 | 17.5 ± 0.4 | 17.5 ± 0.6 | 17.4 ± 0.7 |
| ***S*_c_/*S*_mes_** | 0.92 ± 0.03 | 0.93 ± 0.01 | 0.93 ± 0.02 | 0.92 ± 0.01 | 0.92 ± 0.02 |
| **Chloroplasts per m^-2^ mesophyll (10^9^)** | 65.7 ± 6.5 | 66.3 ± 6.3 | 66.4 ± 4.7 | 66.1 ± 5.8 | 65.9 ± 4.6 |
| **Chloroplast size (mm^2^)** | 10.2 ± 2.9 | 10.2 ± 3.4 | 10.3 ± 4.5 | 10.2 ± 3.5 | 10.3 ± 4.7 |
| **Intercellular airspace (% section)** | 13.3 ± 0.7 | 13.4 ± 0.4 | 13.5 ± 0.2 | 13.2 ± 0.3 | 13.3 ± 0.6 |
| **Mesophyll cells**  **(% section)** | 42.5 ± 1.4 | 42.8 ± 1.8 | 42.3 ± 3.8 | 42.4 ± 3.6 | 42.3 ± 2.6 |
| **Epidermis**  **(% section)** | 13.1 ± 1.1 | 13.1 ± 1.4 | 13.1 ± 1.3 | 13.2 ± 1.4 | 13.3 ± 1.7 |
| **Bulliform cells**  **(% section)** | 10.4 ± 0.5 | 10.3 ± 0.6 | 10.5 ± 0.4 | 10.5 ± 0.3 | 10.3 ± 0.2 |
| **Sclerenchyma strands (% section)** | 4.6 ± 0.1 | 4.5± 0.5 | 4.6 ± 0.2 | 4.6 ± 0.4 | 4.7 ± 0.3 |
| **Outer bundle-sheath (% section)** | 9.3 ± 0.7 | 9.3 ± 0.2 | 9.4 ± 0.1 | 9.5 ± 0.6 | 9.6 ± 0.3 |
| **Vascular bundle**  **(% section)** | 6.8 ± 0.5 | 6.6 ± 0.6 | 6.6 ± 0.3 | 6.6 ± 0.4 | 6.5 ± 0.6 |

Values are means ± SD of all lines for three biological replicates in every group.
